# Air Travel and the Spread of Influenza: Important Caveats

**DOI:** 10.1371/journal.pmed.0030503

**Published:** 2006-11-28

**Authors:** Cécile Viboud, Mark A Miller, Bryan T Grenfell, Ottar N Bjørnstad, Lone Simonsen

**Affiliations:** Fogarty International Center, National Institutes of Health Bethesda, Maryland, United States of America; Center for Infectious Diseases Dynamics, Pennsylvania State University University Park, Pennsylvania, United States of America Fogarty International Center, National Institutes of Health Bethesda, Maryland, United States of America; National Institute of Allergy and Infectious Diseases, National Institutes of Health Bethesda, Maryland, United States of America

While air travel contributes to the spread of influenza epidemics, the magnitude of impact is not clear compared to other factors—a crucial issue when considering a flight ban in the context of pandemic planning. Recent modeling efforts simulating the spread of pandemic influenza have concluded that such an intervention would matter little relative to other interventions [1–3]. But this assessment has now been challenged by an observational study of influenza in the winter following the post-9/11/2001 depression in air traffic. Brownstein and colleagues' study published in the September issue of *PLoS Medicine* [4] correlates variations in air traffic volume with patterns of timing and spread in influenza epidemics, based on United States mortality data from nine epidemic seasons between 1996 and 2005. While we find the study interesting, we have identified several important caveats and question the robustness of the conclusions.

The core of this study's results lies in the observation that the 2001–2002 influenza epidemic immediately following 9/11 was late in the season and peaked in March (week of year 11), whereas the eight surrounding epidemics peaked between the end of December and the end of February (week of year 52 to 9). The authors attribute this delay to the 27% decline in air traffic that followed 9/11.

**Figure 1:**
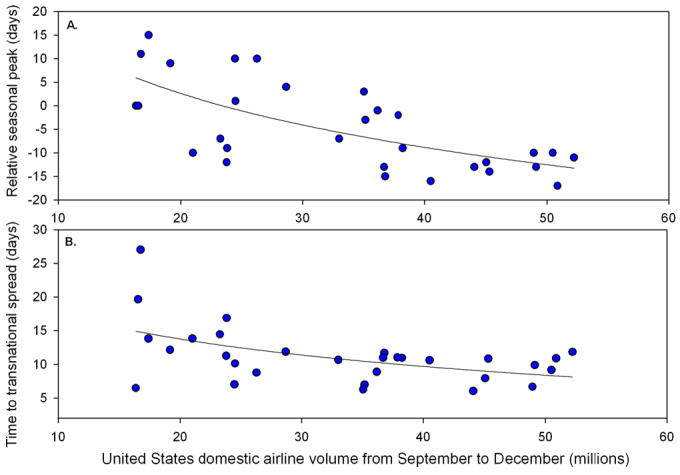
Patterns of Timing (A) and Spread (B) of 30 Influenza Epidemics in the US, Together with Trends in Air Travel Statistics Influenza patterns are based on weekly national vital statistics from 1972 to 2002 [5]. Air travel statistics represent the annual number of domestic and international passengers on US air carriers (scheduled flights, secondary y-axis) [7]. (A) Time series of timing of national peaks of influenza mortality. The 2001–2002 epidemic following 9/11 peaked in March, and so did two epidemics in the 1970s and one in the 1990s. (B) Time series of rate of spread between US states. The rate of spread of the 2001–2002 epidemic following 9/11 is comparable to that of other epidemics. Rate of spread as calculated in [5], based on the timing of epidemic peaks in each state.

Given the complexities of influenza virus subtype cycling and antigenic drift [5,6], it is essential to consider longer-term disease data spanning much more than nine years to interpret the “lateness” of the 2001–2002 epidemic. Using US national vital statistics data covering 30 winters from 1972 to 2002 [5], we identified four epidemics peaking in the month of March (13%), including the 2001–2002 epidemic following 9/11, but also two epidemics in the 1970s and the more recent 1991–1992 epidemic (Figure 1A). Furthermore, the average timing of influenza epidemics has not changed between 1972 and 2002—despite a concurrent and steady increase in air traffic volume by over 300% (Figure 1A) [7]. Indeed, during the earlier part of the last century when air traffic was minimal, influenza epidemics rapidly circulated around the world. Moreover, real-time influenza virus surveillance data from the US Centers for Disease Control and Prevention [8] show that last winter's (2005–2006) epidemic was even more delayed than the epidemic following 9/11, despite a 20% increase in air passenger traffic compared to the situation before 9/11 [7]. Clearly, late-season influenza epidemics have occurred and are still occurring even in the absence of restrictions on air travel. Hence a longer time perspective, with observations from both prior and more recent data, challenges this study's conclusions.

In addition to comparing the timing of influenza epidemics across different seasons, Brownstein et al. analyzed the rate of disease spread among US administrative regions for their nine seasons of interest (1996–2005). In our previous work, we estimated the rate of influenza spread among all US states for 30 consecutive seasons (1972–2002) [5]. Our analysis shows that the epidemic following 9/11 spread at a rate comparable to other epidemics (Figure 1B), even after adjusting for the subtype of circulating viruses [5]. To increase our understanding of the spread of influenza, it is essential to quantify the relative importance of different modes of transportation. As an example, our recent study considered multiple modes of transportation (including air travel) and identified travel to and from work as a key determinant of the regional spread of epidemics [5].

In conclusion, Brownstein and colleagues' analysis of the “natural experiment” of the post-9/11 season is innovative and ingenious—but in and of itself could not demonstrate a robust association or a causal link between the decrease in air traffic and delayed timing of influenza epidemics. Even if there in fact had been a delay as hypothesized, the study lacked power to address the hypothesis, because this single “natural experiment” was set in a background of considerable variability in influenza epidemic patterns. Extrapolations from the study's findings predict that a flight ban could delay a pandemic by two months [9]—but we have shown here that this prediction is not supported by the analysis of more extensive disease data and transportation statistics. It is also unclear how a “natural experiment” conducted in the inter-pandemic period is applicable to a pandemic situation, where novel influenza viruses have higher transmissibility and circulate in fully susceptible populations, and may cause different age-patterns of transmission [10]. While Brownstein and colleagues' study represents an intriguing starting point, this study alone does not provide the critical quantitative evidence needed to evaluate the impact of travel restrictions on future pandemics.
