# Correction: Air Travel and the Spread of Influenza: Important Caveats

**DOI:** 10.1371/journal.pmed.0040032

**Published:** 2007-01-30

**Authors:** Cécile Viboud, Mark A Miller, Bryan T Grenfell, Ottar N Bjørnstad, Lone Simonsen

In *PLoS Medicine*, volume 3, issue 11, doi:10.1371/journal.pmed.0030503:

Figure 1 from this correspondence was transposed with Figure 1 from the related correspondence by Brownstein and colleagues (“Air Travel and the Spread of Influenza: Authors' Reply” doi:10.1371/journal.pmed.0030502).

The legend published with Figure 1 (“Patterns of Timing (A) and Spread (B) of 30 Influenza sEpidemics in the US, Together with Trends in Air Travel Statistics”) is correct. However, the figure itself should have appeared as shown below.

**Figure 1:**
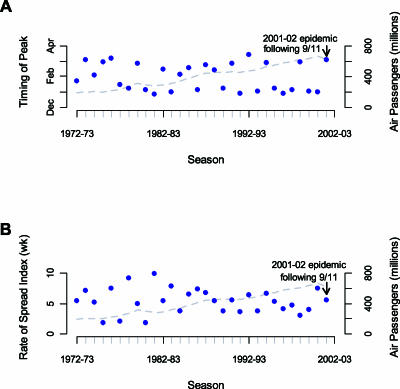
Patterns of Timing (A) and Spread (B) of 30 Influenza Epidemics in the US, Together with Trends in Air Travel Statistics

